# Case Report: A case of lymph node metastatic cancer of unknown primary with elevated alpha-fetoprotein achieving long-term survival

**DOI:** 10.3389/fonc.2025.1724466

**Published:** 2026-01-05

**Authors:** Yan Wang, Chao-Qun Wang, Bi-Fei Huang

**Affiliations:** 1 Department of Medical Oncology, Affiliated Dongyang Hospital of Wenzhou Medical University, Dongyang, Zhejiang, China; 2 Department of Pathology, Affiliated Dongyang Hospital of Wenzhou Medical University, Dongyang, Zhejiang, China

**Keywords:** alpha-fetoprotein, carcinoma of unknown primary, case report, metastasis, survival

## Abstract

**Introduction:**

Carcinoma of unknown primary (CUP) is typically associated with a poor prognosis, with a median survival of less than one year. This article reports a rare case of an alpha-fetoprotein (AFP)-positive CUP patient who achieved a long-term survival of nearly five years, whose diagnostic and therapeutic course offers significant clinical insights.

**Case presentation:**

A 67-year-old male patient presented with left neck lymphadenopathy. A comprehensive workup revealed no primary site, leading to a diagnosis of CUP. Although subsequent immunohistochemistry classified the case into the unfavorable prognostic subgroup, an individualized treatment strategy including three-drug combination chemotherapy based on the clinical suspicion of gastric cancer, targeted therapy with Apatinib, and localized radiotherapy, resulted in a remarkably long overall survival of 58 months. Serum AFP levels closely correlated with disease activity throughout the clinical course.

**Conclusions:**

This case demonstrates that for CUP confined to lymph nodes, active and precisely reasoned individualized treatment can significantly improve prognosis, even in cases categorized as having an unfavorable prognosis. Furthermore, it underscores the necessity of immunohistochemistry for the accurate diagnosis of CUP and suggests that promoting precision medicine strategies may improve outcomes for these patients.

## 1 Introduction

Carcinoma of Unknown Primary (CUP) refers to a heterogeneous group of pathologically confirmed metastatic malignancies whose primary anatomical site cannot be identified through conventional examinations. In the era of targeted therapies, accurate histopathological and molecular classification, including epigenetic profiling, is essential for individualized treatment (1). CUP accounts for approximately 2%–5% of all malignant tumors (2). Its pathogenesis remains unclear, and the prognosis is generally poor, with a median survival often less than one year, posing significant challenges in clinical diagnosis and treatment (3, 4). However, novel favorable subsets, including colorectal-like, lung-like, and renal-like CUP, have been recognized, which can guide site-specific treatment strategies (5).

Metastases confined to lymph nodes are relatively uncommon in CUP, comprising about 10% of cases (6). The most frequent histological type of CUP is adenocarcinoma, while others such as squamous cell carcinoma and neuroendocrine carcinoma are rare (4, 7). Additionally, approximately 3% of melanomas present as melanoma of unknown primary (MUP), which may have better outcomes and enhanced immunogenicity, potentially leading to improved response to immunotherapy (8). Compared to cases with widespread metastasis or visceral involvement, patients with nodal-limited disease typically have a relatively better prognosis: the 12-month survival rate for extranodal CUP is 17%, with a median survival of 3 months; when metastasis is limited to lymph nodes, the 12-month survival rate is 41%, with a median survival of 8 months (4). However, due to the occult nature of the primary site and diverse histological presentations, accurate diagnosis and classification heavily rely on pathological morphology combined with ancillary techniques such as immunohistochemistry. Treatment strategies often require individualized reasoning.

Currently, platinum-based doublet chemotherapy is the standard treatment for unfavorable-prognosis CUP, but its efficacy is limited, and survival benefits are difficult to improve (9). Therefore, exploring effective individualized treatment strategies to enhance patient outcomes has become a key focus in clinical practice. Furthermore, chromosomal instability is not frequent in CUP, which may favor immune checkpoint inhibitors (ICI), although individual gene alterations can cause immune evasion and ICI resistance, requiring further investigation (10). This article reports a rare case of unfavorable-prognosis CUP, confirmed by immunohistochemistry, which presented with long-term nodal confinement and alpha-fetoprotein (AFP) positivity. Through personalized comprehensive treatment based on precise clinical reasoning, the patient achieved a survival of 58 months. This case aims to provide valuable insights for the clinical management of CUP.

## 2 Case description

**Figure 1:**
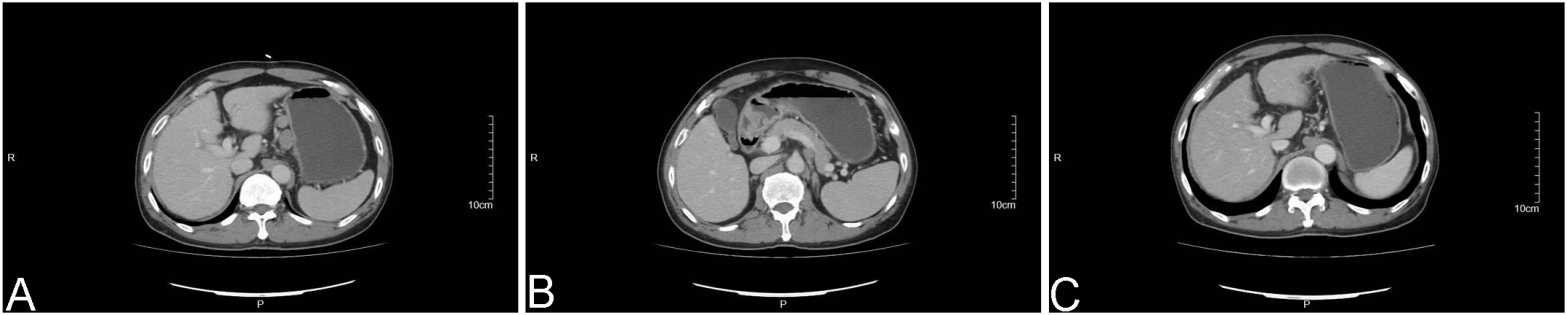
Computed tomography (CT) images: **(A)** An abdominal CT scan from August 2013 reveals enlarged lymph nodes adjacent to the lesser curvature of the stomach; **(B)** An abdominal CT scan from August 2013 shows enlarged lymph nodes in the retroperitoneal area, with no space-occupying lesion identified in the gastric wall; **(C)** An abdominal CT scan from February 2016 demonstrates no evidence of enlarged lymph nodes.

The patient is a 67-year-old male with a history of smoking, alcohol abuse, and alcoholic cirrhosis, and no family history of cancer. On August 23, 2013, he presented with a “small left neck mass accompanied by pain.” Ultrasound examination revealed multiple enlarged lymph nodes in the left supraclavicular area, the largest measuring 2.0 cm × 1.0 cm, with clear borders, homogeneous echogenicity, and moderately enhanced vascularity within the mass. Computed tomography (CT) scans of the chest, abdomen, and pelvis, as well as an abdominal magnetic resonance imaging (MRI), showed multiple enlarged lymph nodes in the gastric antrum, along the lesser curvature, and in the retroperitoneal area (**Figures 1A, B**). Positron emission tomography-computed tomography (PET-CT) results indicated enlarged lymph nodes deep to the left sternocleidomastoid muscle and in the left clavicular region, with a maximum diameter of 1.71 cm and SUVmax of 9.2, suggestive of metastasis; multiple enlarged lymph nodes along the lesser curvature of the stomach, around the pancreas, and in the retroperitoneum, with a maximum diameter of 3.36 cm and SUVmax of 10.46, also suggestive of metastasis. Tumor marker tests revealed an AFP level of 185.4 ng/ml, while squamous cell carcinoma-related antigen, cancer antigen 125 (CA125), and prostate-specific antigen (PSA) were within normal ranges. Gastroscopic biopsy pathology showed moderate to severe superficial inflammation with erosion. The patient reported no gastrointestinal symptoms such as early satiety, nausea, vomiting, abdominal pain, or melena. A colonoscopy was not tolerated and therefore could not be completed. A percutaneous biopsy of the left supraclavicular lymph node was performed in our outpatient department, and pathology confirmed metastatic carcinoma, primarily suggestive of metastatic squamous cell carcinoma. However, immunohistochemical testing was not performed. Clinical diagnosis: Metastatic squamous cell carcinoma in the lymph nodes, with an unknown primary site.

The patient received six cycles of chemotherapy from September 6, 2013, to January 20, 2014, according to the regimen: Paclitaxel 100mg d1, d8; Cisplatin 40mg d1-d3; Fluorouracil 4000mg q72h, repeated every 3 weeks. A follow-up neck lymph node ultrasound after the first cycle showed no lymph nodes larger than 1 cm, and AFP decreased to normal levels (2.9 ng/ml). Tumor assessment after the 2nd and 4th cycles indicated partial response (PR), and after 6 cycles, a complete response (CR) was achieved, with AFP remaining normal. The patient underwent regular follow-up after completing chemotherapy.

In December 2014, a follow-up ultrasound revealed enlarged lymph nodes in zones IV and V of the left neck, and AFP was elevated to 54.1 ng/ml, suggesting local tumor recurrence. On December 12, 2014, the patient underwent “Resection of a deep mass in the maxillofacial and cervical region” at another hospital. Postoperative pathology indicated metastatic poorly differentiated carcinoma in the lymph nodes, and clinical investigation of the liver and gastrointestinal tract was recommended. (Immunohistochemistry: P63 -, PSA -, CDX2 +, CK7 -, CK20 -, TTF-1 -, CgA partially +, Syn partially +, AFP partially +). Chest CT and abdominal MRI showed no lesions or enlarged lymph nodes. Routine gastroscopy and endoscopic ultrasound revealed no suspicious lesions; gastric biopsy pathology showed mild chronic superficial inflammation of the mucosa. Local radiotherapy was recommended but refused by the patient.

As the patient could not tolerate the original chemotherapy regimen, he received three cycles of S-1 (60mg twice daily, days 1-14) from December 20, 2014, to January 31, 2015, but no shrinkage of the neck lymph nodes was observed. A follow-up ultrasound in March 2015 showed continued enlargement of the left neck lymph nodes, and AFP increased to 176.4 ng/ml, indicating disease progression.

Targeted therapy with Apatinib Mesylate (850mg once daily) was initiated on March 23, 2015. After one week, the neck lymph nodes had shrunk compared to pre-treatment size, and by April 8, 2015, AFP had decreased to 71.46 ng/ml. However, the patient experienced side effects including fatigue, poor appetite, and thrombocytopenia, leading to a gradual dose reduction to 250mg once daily. Regular follow-up with neck ultrasound and abdominal MRI during this period showed stable disease (SD) in the neck lymph nodes and no new lesions elsewhere.

In January 2016, the patient was found to have increased and enlarged left neck lymph nodes. An abdominal CT in February 2016 showed no abnormal tumor lesions (**Figure 1C**), but a chest CT revealed multiple enlarged lymph nodes in both sides of the neck, supraclavicular areas, and the mediastinum. Local radiotherapy targeting the neck, supraclavicular, and mediastinal lymph nodes began on February 29, 2016, using IMRT: P-GTV 66Gy/30f.

In April 2016, the patient developed new enlarged left axillary lymph nodes but received no specific treatment. By December 4, 2017, AFP had risen significantly to 2028 ng/ml. A follow-up abdominal CT and neck ultrasound on December 9, 2017, revealed multiple enlarged lymph nodes in the neck and retroperitoneum. A percutaneous biopsy of the left axillary lymph node was performed at our institution. The pathological diagnosis suggested metastatic carcinoma, with immunohistochemistry favoring adenocarcinoma, likely of gastrointestinal origin. (Immunohistochemistry: CAM5.2 +, P63 -, CDX2 +, CK7 rare cells +, CK20 rare cells +, HER-2 1+, TTF-1 -, Napsin-A -, ER -, PR -, Ki-67 approximately 60% +).

Radiotherapy targeting the left axillary lymph nodes was administered on April 30, 2018, and the procedure was well-tolerated. Subsequently, the patient received only palliative supportive care without further systemic anti-tumor therapy until his death on July 7, 2018.

**Figure 2:**
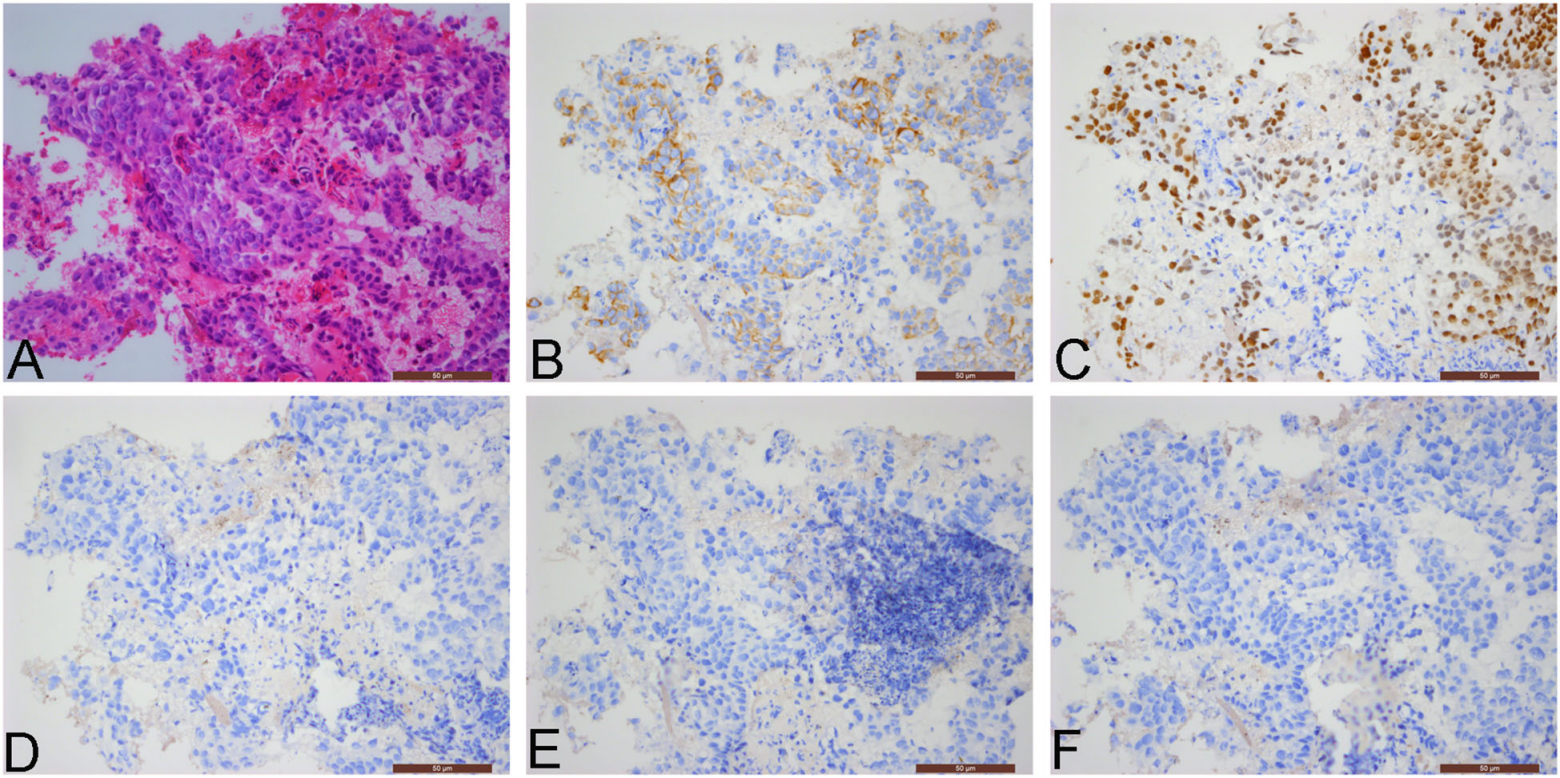
Pathological images from the percutaneous biopsy of the left supraclavicular lymph node (August 2013; 200× magnification): **(A)** Hematoxylin and eosin (H&E) staining shows poorly differentiated carcinoma; **(B)** Immunohistochemical staining for CAM5.2 is positive, with cytoplasmic staining in tumor cells; **(C)** Immunohistochemical staining for CDX2 is positive, with nuclear staining in tumor cells; **(D)** Immunohistochemical staining for CK7 is negative; **(E)** Immunohistochemical staining for CK20 is negative; **(F)** Immunohistochemical staining for P40 is negative.

**Figure 3:**
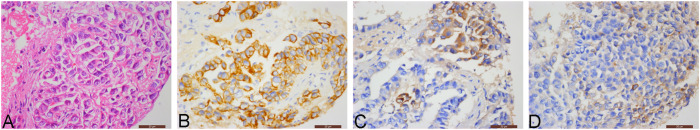
Pathological images from the percutaneous biopsy of the left axillary lymph node (December 2017; 400× magnification): **(A)** Hematoxylin and eosin (H&E) staining shows poorly differentiated carcinoma; **(B)** Immunohistochemical staining for CAM5.2 is positive, with cytoplasmic staining in tumor cells; **(C, D)** Immunohistochemical staining for AFP is partially positive, with cytoplasmic staining in tumor cells.

Since immunohistochemical staining was not initially performed on the August 2013 percutaneous biopsy specimen of the left supraclavicular lymph node, a retrospective immunohistochemical analysis was conducted in August 2025. The findings were as follows: CAM5.2 +, CDX2 +, CK7 -, CK20 -, P63 -, P40 -, CK5/6 -, AFP -. Upon pathologic re-evaluation of the slides, the diagnosis was amended to metastatic adenocarcinoma (**Figure 2**), most likely of gastrointestinal origin. Furthermore, given that AFP immunohistochemistry had not been conducted on the December 2017 left axillary lymph node puncture specimen, AFP staining was performed retrospectively on the same punctured tissue. This additional staining demonstrated AFP positivity in a portion of the tumor cells (**Figure 3**).

## 3 Discussion

We report a case of AFP-producing metastatic lymph node carcinoma with a long survival period, highly suspected to be of gastrointestinal origin. As multiple imaging examinations, including CT, MRI, PET-CT, two gastroscopies, and one endoscopic ultrasound, failed to identify the primary lesion, the case was diagnosed as metastatic lymph node carcinoma, a CUP. Although CUP accounts for 2%-5% of all cancers (2), and its incidence is declining due to advancements in imaging and molecular diagnostic techniques (7), the diagnostic challenges and poor prognosis associated with CUP warrant clinical attention. According to the literature, the median survival time for CUP is less than one year (3, 4).

The most common histological type of CUP is adenocarcinoma, along with other less frequent types such as squamous cell carcinoma and neuroendocrine carcinoma, which collectively account for a small proportion of cases (4, 7). Additionally, sarcoma of unknown primary (SUP) represents another rare entity characterized by considerable histologic heterogeneity, which complicates diagnostic confirmation. The SUP phenomenon may be theoretically explained by a primary tumor that is too small to be detected or due to an inadequate diagnostic workup. It has been reported that two-thirds of SUP patients present with more than one metastatic site (11). The liver is the most frequent site of metastasis, followed by the respiratory system, lymph nodes, and abdominal cavity (2). Metastatic disease confined solely to lymph nodes occurs in only about 10% of cases (6). The European Society for Medical Oncology (ESMO) guidelines classify CUP into favorable and unfavorable prognostic subgroups based on the site of involvement, histology, and immunohistochemical profile (9). The favorable subgroup comprises approximately 20% of cases. Adenocarcinomas with a colorectal immunophenotype (CK7 -, CK20 +, CDX2 +) fall into this category, and these patients are recommended to receive site-directed therapy targeting the suspected primary origin for better outcomes. The remaining 80% of patients belong to the unfavorable prognostic subgroup. A retrospective study focusing on unfavorable subgroup adenocarcinomas with gastrointestinal features (CK7+/CK20-/any CDX2 OR CK7-/CK20+/CDX2- OR CK7-/CK20-/CDX2+) reported a median overall survival (OS) of just 11.8 months. In the subgroup that received chemotherapy, the median progression-free survival (PFS) was 6.1 months, and the median OS was 12.6 months (12). This underscores the critical role of immunohistochemistry in the diagnosis, classification, treatment guidance, and prognosis of CUP. Based on the immunohistochemistry results, the patient in this report belonged to the unfavorable prognostic subgroup. The initial pathological diagnosis from the August 2013 left supraclavicular lymph node biopsy suggested metastatic carcinoma, primarily considered metastatic squamous cell carcinoma. However, the absence of further relevant immunohistochemical testing at that time led to a misdiagnosis. The definitive diagnosis of an adenocarcinoma likely of gastrointestinal origin was only established retrospectively in August 2025 following immunohistochemistry analysis. This case highlights the importance of pathologists routinely performing immunohistochemical staining on metastatic tumors of unknown origin in lymph nodes to provide clinicians with as much information as possible regarding the potential primary site.

Despite the absence of a identified primary site after comprehensive investigations and the lack of gastrointestinal symptoms such as dysphagia, nausea, vomiting, hematemesis, melena, abdominal pain, or distension, clinicians maintained a high suspicion for gastric cancer with lymph node metastasis. This suspicion was primarily based on the distribution of the enlarged lymph nodes, which was characteristic of typical gastric cancer metastasis patterns–involving the gastric antrum, hepatogastric space, retroperitoneum, and left supraclavicular area. The elevated AFP level was another significant contributing factor. In clinical practice, AFP-producing carcinoma (AFPGC) is often defined as gastric cancer with a serum AFP level >20 ng/ml or immunohistochemical evidence of AFP positivity (13). Primary gastric cancer exhibiting morphological features of hepatocellular differentiation is specifically classified as hepatoid adenocarcinoma (HAS) (13). While HAS can occur in various organs like the colorectum and lung, it most frequently arises in the stomach (14, 15). Some scholars hypothesize that AFPGC might originate from conventional adenocarcinoma cells in the gastric mucosa. During tumor progression, these cells may undergo reverse/aberrant differentiation towards pluripotent embryonic stem cell-like states, leading to various pathological subtypes, including HAS, and concurrently acquiring the ability to secrete AFP (13). In our patient, the initial serum AFP was 185.4 ng/ml. Fluctuations in AFP levels correlated with treatment response and disease progression. Histological examination of lymph node biopsies on three separate occasions consistently showed poorly differentiated carcinoma, with two of the samples demonstrating focal positivity for AFP on immunohistochemistry. Notably, the patient never developed liver metastases throughout the disease course. Elevated AFP is rarely reported in CUP cases presenting with isolated lymph node metastasis. Therefore, this case was highly suggestive of AFPGC. AFPGC is typically characterized by rapid progression, high malignancy, poorer prognosis compared to conventional gastric cancer, and a high incidence of liver metastasis, with a reported median OS of 13.9 months (16). Interestingly, our patient’s disease remained confined to the lymph nodes without extra-nodal metastasis (e.g., to liver, peritoneum, or lungs) and achieved a nearly 5-year overall survival, which was unexpected. This unique clinical course leads us to speculate that CUP might represent a distinct disease entity with biological behaviors different from those of its suspected primary tumor.

Currently, platinum-based doublet chemotherapy remains the recommended standard treatment for CUP with an unfavorable prognosis, although no specific regimen has been definitively established as superior (12). Rational clinical reasoning by physicians is crucial in formulating treatment plans. It is noteworthy that predictive biomarkers for ICI are present in a subset of CUP patients; for instance, programmed death-ligand 1 (PD-L1) expression is found on ≥5% of cancer cells in 22.5% of cases (≥1% in 34%) and on tumor-infiltrating lymphocytes in 58.7%, while microsatellite instability-high (MSI-H) accounts for 1.8% and tumor mutational burden (TMB) ≥17 mutations per megabase occurs in 11.8%. Although these biomarkers are not yet validated in CUP, patients with TMB >10 mutations per megabase tend to show better outcomes when treated with ICI (17). In this case, although the initial pathological findings primarily suggested squamous cell carcinoma, the clinicians had a high suspicion of gastric cancer. Given the patient’s Eastern Cooperative Oncology Group (ECOG) performance status of 0, fluorouracil was added to the first-line platinum-based regimen. This approach ultimately resulted in a clinical complete response and achieved a PFS of 14 months. Subsequent treatments were also selected following gastric cancer treatment guidelines (18). Although second-line therapy was limited to the single-agent S-1 due to declining performance status, yielding suboptimal results, third-line treatment with the anti-angiogenic agent Apatinib achieved a PFS of nearly 9 months, superior to the 3.5 months reported in the literature (19). The selection of apatinib was based on a clinical trial in which apatinib monotherapy demonstrated improved median PFS and OS compared to placebo in third-line advanced gastric cancer (20), leading to its approval in China for this indication in 2014. This case suggests that for patients with a good ECOG score, three-drug combination chemotherapy may offer better efficacy. After disease progression in January 2016, this patient received only localized radiotherapy targeting the lymph nodes without further systemic anti-tumor therapy. Nevertheless, he survived for an additional 30 months, resulting in a total OS of 58 months, which was significantly longer than the median OS of 8 months reported in the literature for nodal-confined CUP (4).

Recent advances in genetic testing have revealed that nearly all CUP samples harbor at least one clinically significant genetic mutation that may influence prognosis and enable personalized treatment. Comprehensive genomic profiling could potentially expand treatment options for CUP patients, thereby addressing the current limitations of limited therapeutic strategies and poor prognosis (21). A Chinese single-center clinical trial (Fudan CUP-001) utilized a 90-gene expression assay to predict the tissue of origin in CUP (22). The enrolled study population consisted entirely of Asian patients, with histological types including adenocarcinoma, squamous cell carcinoma, and poorly differentiated carcinoma. Approximately 50% of the participants had metastases confined solely to lymph nodes. Patients who received site-specific therapy based on the predicted origin achieved a significantly longer median PFS compared to those receiving empirical chemotherapy (9.6 months *vs*. 6.6 months). Notably, in the subgroup with disease confined to lymph nodes, the median PFS was 12.5 months and 7.4 months, respectively (22). A randomized phase II multicenter study (CUPISCO) investigated patients with CUP of adenocarcinoma or poorly differentiated carcinoma (23). All patients received first-line platinum-based chemotherapy. Among those who achieved disease control after three cycles, patients with identifiable targetable mutations who subsequently received targeted therapy achieved a median PFS of 8.1 months, compared to 4.7 months in those who continued with chemotherapy. This suggests that combining gene-directed therapy with chemotherapy may improve first-line PFS and potentially enhance outcomes, though confirmation from larger studies is still awaited (23). It is important to note that current studies comparing site-specific therapy and empiric chemotherapy face significant limitations, including patient accrual challenges, study design constraints, heterogeneity among CUP classifiers and incomparable therapeutic regimens. Recent assessments of CUP literature recommend two comprehensive clinical trial designs, a visionary and a pragmatic approach, both amenable to implementing advanced diagnostics and therapies to improve research quality and patient prognosis (24). However, the high cost of comprehensive genomic profiling may limit its routine clinical application, though it is noteworthy that next-generation sequencing (NGS) is often covered by insurance in the United States. We recommend that NGS testing should be pursued to guide more precise targeted therapy for patients who can access and consent to such profiling.

## 4 Conclusion

This article reports a rare case of CUP with long-term survival. The patient presented with AFP-producing metastatic lymph node carcinoma, highly suspected to be of gastrointestinal origin, although the primary site remained unidentified throughout the course. Despite being classified into the unfavorable prognostic subgroup based on immunohistochemistry results and an initial misdiagnosis due to the lack of immunohistochemical testing, the patient achieved an OS of nearly five years–significantly exceeding the median survival for CUP–through a personalized treatment strategy. This regimen included fluorouracil-containing chemotherapy based on the clinical suspicion of gastric cancer, anti-angiogenic targeted therapy, and localized radiotherapy. This case suggests that for CUP patients with disease confined to lymph nodes, active and precisely reasoned individualized treatment may improve prognosis. It also underscores the critical role of immunohistochemistry and genetic testing in the diagnosis and management of CUP. Promoting precision medicine strategies is essential to improve outcomes for such patients in the future.

## Data Availability

The original contributions presented in the study are included in the article/supplementary material. Further inquiries can be directed to the corresponding author.
